# Apixaban plasma concentrations before and after catheter ablation for atrial fibrillation

**DOI:** 10.1371/journal.pone.0308022

**Published:** 2024-07-31

**Authors:** Rachel Aakerøy, Jan Pål Loennechen, Roar Dyrkorn, Stian Lydersen, Arne Helland, Olav Spigset

**Affiliations:** 1 Department of Clinical and Molecular Medicine, Faculty of Medicine and Health Sciences, Norwegian University of Science and Technology, Trondheim, Norway; 2 Department of Clinical Pharmacology, St. Olav University Hospital, Trondheim, Norway; 3 Clinic of Cardiology, St. Olav University Hospital, Trondheim, Norway; 4 Department of Circulation and Medical Imaging, Norwegian University of Science and Technology (NTNU), Trondheim, Norway; 5 Regional Centre for Child and Youth Mental Health and Child Welfare, Department of Mental Health, Faculty of Medicine and Health Sciences, Norwegian University of Science and Technology, Trondheim, Norway; University of Liverpool, UNITED KINGDOM OF GREAT BRITAIN AND NORTHERN IRELAND

## Abstract

**Background:**

Catheter ablation in patients with atrial fibrillation is associated with a transient increase in thromboembolic risk and adequate anticoagulation is highly important. When patients are anticoagulated with apixaban, monitoring of plasma concentrations of the drug is not routinely performed. This study aimed to assess the influence of clinical patient characteristics, concomitant drug treatment and self-reported adherence on apixaban concentrations, and to describe the intra- and inter-individual variability in apixaban concentrations in this group of patients.

Method

Apixaban concentrations from 141 patients were measured in plasma one week before ablation and two, six and ten weeks after ablation, employing ultra-high performance liquid chromatography coupled with tandem mass spectrometry. In samples not obtained at trough, apixaban concentrations were adjusted to trough levels. Self-reported adherence was registered by means of the 8-item Morisky Medication Adherence Scale before and after ablation.

**Results:**

There were statistically significant, positive correlations between apixaban concentrations and increased age, female sex, lower glomerular filtration rate, higher CHA_2_DS_2_-VASc score, use of cytochrome P450 3A4 and/or p-glycoprotein inhibitors, and use of amiodarone. Self-reported adherence was generally high. The mean intra-individual and inter-individual coefficients of variation were 29% and 49%, respectively.

**Conclusion:**

In patients undergoing catheter ablation for atrial fibrillation, age, sex, renal function, interacting drugs and cerebrovascular risk profile were all associated with altered plasma apixaban concentration. In this group of patients with a generally high self-reported adherence, intra-individual variability was modest, but the inter-individual variability was substantial, and similar to those previously reported in other patient apixaban-treated populations. If a therapeutic concentration range is established, there might be a need for a more flexible approach to apixaban dosing, guided by therapeutic drug monitoring.

## Introduction

Atrial fibrillation (AF), a common arrhythmia worldwide, is a major risk factor for ischemic stroke (IS) [1]. Lifetime risk of developing AF has been shown to increase with age from about 1:4 at age 40 years and older to approximately 1:3 in patients older than 55 years of age [1–3]. Catheter ablation has become a commonly used procedure to treat AF. As AF ablation results in a transiently increased thromboembolic risk [4], anticoagulation is initiated at least 3 weeks prior to ablation and is continued for at least two months after the procedure [5].

Earlier studies have shown that apixaban therapy is a safe and effective alternative to Vitamin K antagonists in preventing stroke in patients undergoing ablation for AF [6, 7]. Apixaban is rapidly absorbed, with maximum concentration occurring 3–4 hours after oral administration. Oral bioavailability is around 50%. It is predominantly metabolized in the liver by cytochrome P450 3A4 (CYP3A4) and is also a substrate for the membrane transporter P-glycoprotein (P-gp). Approximately 30% of total apixaban clearance occurs via renal excretion. The mean elimination half-life is 12 hours [8].

In patients treated with vitamin K antagonists, the intensity of anticoagulation as well as adherence to therapy can be monitored by means of the international normalized ratio (INR). In contrast, routine therapeutic drug monitoring (TDM) of apixaban concentrations is generally not performed except in a few special circumstances, for instance in patients with impaired renal function, extremes of body weight and bleeding [9]. Thus, this lack of monitoring may result in suboptimal adherence going unnoticed. Due to the short half-life of apixaban, theoretically as little as two consecutive missed doses can potentially increase the risk of ischemic stroke. Previous studies have shown that decreased adherence to direct oral anticoagulants (DOACs) is indeed associated with an increased risk of stroke [10, 11]. To our knowledge, no previous studies have measured plasma concentrations of apixaban with ultra-high performance liquid chromatography coupled with tandem mass spectrometry (UHPLC-MS-MS), considered to be the gold standard for drug concentration analysis [9], and related drug concentrations to self-reported adherence data in patients undergoing ablation for AF.

This study aimed to assess the intra- and inter-individual variability in apixaban concentrations in patients undergoing catheter ablation for AF, as well as the influence of age, sex, renal function, CHA_2_DS_2_-VASc score, concomitant drug treatment and self-reported adherence on apixaban concentrations.

## Materials and methods

### Study population

Patients scheduled to undergo catheter ablation for AF at the Cardiology Clinic at St. Olav University Hospital, Trondheim, Norway and who were being treated with apixaban or rivaroxaban were eligible for inclusion. Patients were included consecutively from 6^th^ of April 2021 until the 8^th^ of June 2022. An invitation to participate, with detailed information about the study, was sent by mail. Patients willing to participate returned a signed consent form. In total, 402 patients were asked to participate, of whom 164 consented. Patients using rivaroxaban were too few to give meaningful data. After excluding those treated with rivaroxaban (n = 19) and those lost due to administrative errors (n = 4), 141 patients were finally included in the analyses.

The study was approved by the Regional Committee for Medical Research Ethics in Mid Norway (registration number 2018/1815/REK Midt) and is registered in the European Union Electronic Register of Post-Authorisation Studies (EU PAS Register number EUPAS105053). The Norwegian Medicines Agency was consulted, and they concluded that the study was not defined as a clinical study according to Norwegian legislation, and that further approval from the drug authorities was not required.

### Clinical variables

Clinical information was retrieved from medical records. Variables included those needed to calculate the CHA_2_DS_2_-VASc score, which predicts risk of stroke in patients with AF [12]. Estimated glomerular filtration rate (eGFR; mL/min per 1.73 m^2^) was obtained automatically from the laboratory, based on the CKD-EPI formula [13]. Absolute GFR (mL/min) was calculated, taking into consideration the patients’ body weight and height, and is the measure of renal function used in this article. Potentially interacting concomitant drugs were classified as CYP3A4 and/or P-gp inducers or inhibitors based on the overview of such drugs listed in a comprehensive guideline [9].

### Blood sampling and analysis of apixaban

Patients were asked to contact their general practitioner to have blood samples drawn for the analysis of apixaban. These samples were collected one week before ablation (“baseline”) and at two, six and ten weeks after ablation. Samples were collected in 3.5 mL Greiner Vacuette® citrate tubes with 3.8% sodium citrate. All tubes were centrifuged at 2000 g for 10 minutes before plasma was pipetted off and analyzed at the Department of Clinical Pharmacology at St. Olav University Hospital.

All samples were analyzed with UHPLC-MS-MS with a standard range of 5–800 nmol/L (2.3–368 ng/mL) as described in detail previously [14]. Time of sampling after last intake of medication varied from 5 hours to 16 hours. Only eight plasma samples were drawn less than 9 hours after last dose. No samples were obtained close to the assumed concentration maximum, i.e., less than 4 hours after last dose [15]. The observed apixaban concentration at *t* hours after intake was converted to an estimated trough 12-hour concentration, according to the following equation:

$$Ct=C\times e^{-\frac{ln2\times \Delta t}{12}}$$

where Ct is the calculated trough concentration level, C is the actual measured concentration, and Δt is the time interval between the time of sampling and the time of achieved trough concentration, i.e. 12 hours after drug intake. A mean elimination half-life of 12 hours was used [15], as indicated in the denominator of the exponent. The molarity to mass conversion factor (e.g. from nmol/L to ng/mL) for apixaban is 0.460.

## Medication adherence

Medication adherence was assessed by means of the validated 8-item Morisky Medication Adherence Scale (MMAS-8) [16, 17]. The MMAS-8 scale, content, name, and trademarks are protected by US copyright and trademark laws (©MMAS www.adherence.cc). Seven out of eight items in the MMAS-8 have a dichotomous response option while the last item has a 5-point Likert response. A score of 8 points is defined as high adherence, 6 to <8 points as medium adherence and <6 as low adherence. The first MMAS-8 scale was distributed together with the invitation to participate in the study. Patients who consented to participate returned the completed scale together with their signed consent form by mail. Eleven weeks after the ablation, a new invitation to fill in the MMAS-8 was sent to the participants. Again, the participants returned their completed MMAS-8 scales by mail.

### Statistical analyses

Descriptive statistics are presented as means with standard deviations for continuous variables and as counts and percentages for categorical variables.

The intra- and inter-individual variability in apixaban concentrations were estimated using a random effect model with apixaban concentration as dependent variable and patient as random effect. We used the restricted maximum likelihood estimator (REML) to avoid downward bias in the variance estimates.

The course of apixaban concentrations over time was described using a linear mixed model with concentration as dependent variable, time point as categorical covariate and patient as random effect. To study the influence of other variables on the apixaban concentration, we used a linear mixed effect model with concentration as dependent variable and time point as categorical covariate, and age, sex, renal function, CHA_2_DS_2_-VASc score and concomitant drug treatment with CYP3A4/P-gp inhibitors and with amiodarone, one at a time, as covariate, and patient as random effect. Normality of residuals was checked by visual inspection of QQ-plots. The effect of adherence as measured by MMAS-8 on the apixaban concentration at baseline and at the end of the study was studied using linear regression with adherence as covariate.

Two-sided *p*-values <0.05 were considered to represent statistical significance. All statistical analyses were performed with IBM SPSS version 29 (IBM, Armonk, NY, USA).

## Results

Demographic and clinical characteristics of the study population at inclusion are shown in Table 1. All patients used an apixaban daily dose of 10 mg (i.e. 5 mg twice daily).

**Table 1:** Demographic and clinical characteristics at baseline of the 141 patients included in the study.

Variable	Value ^a^ (N = 141 ^b^)
**Female**	40 (28%)
**Age (years)**	67.1 (9.6)
**BMI (kg/m** ^**2**^ **)**	27.3 (4.1)
**Serum creatinine concentration (μmol/L)**	83.8(19.0)
**Glomerular filtration rate (mL/min)**	93.3 (23.2)
**Diabetes mellitus**	16 (11%)
**Previous TIA/ischemic stroke**	9 (6%)
**Previous intracranial bleeding**	3 (2%)
**Current smoker**	5 (4%)
**Hypertension**	56 (40%)
**Liver disease** ^**c**^	None
**High alcohol consumption** ^**d**^	2 (1%)
**Coronary artery disease**	18 (13%)
**Peripheral artery disease**	5 (4%)
**Heart failure**	28 (20%)
**CHA**_**2**_ **DS**_**2**_ **-VASc score**	2.2 (1.5)
**Use of CYP3A4 and/ or P-gp inducers** ^**e**^	2 (1%)
**Use of CYP3A4 and/ or P-gp inhibitors** ^**f**^	55 (39%)
**Use of amiodarone**	43 (31%)

Abbreviations: BMI = body mass index; TIA = transient ischemic attack; CHA_2_DS_2_-VASc = a measure of stroke risk; C = congestive heart failure, H = hypertension, A = age, D = diabetes mellitus, S = previous stroke/TIA/thromboembolism, VA = vascular disease, Sc = sex category; CYP3A4 = cytochrome P450 3A4; P-gp = P-glycoprotein

^a^ Numbers are n (%) or mean (standard deviation; SD) as appropriate.

^b^ N = 138 for BMI

^c^ Cirrhosis or steatosis

^d^ High alcohol consumption noted in hospital medical records

^e^ CYP3A4 and/or P-gp inducer in both cases was prednisolone

^f^ CYP3A4 and/or P-gp inhibitors were verapamil, diltiazem, dronedarone and amiodarone

Of the 141 patients, 48 (34%) had a complete set of blood samples with four apixaban concentration measurements. The number of missing samples were 36 at baseline, 35 at two weeks, 38 at six weeks and 47 at ten weeks after ablation.

**Fig 1:**
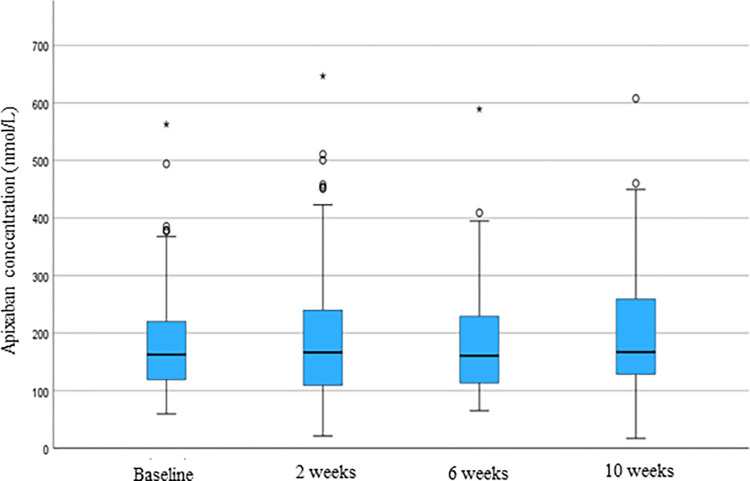
Apixaban plasma trough concentration (nmol/L) at baseline and 2, 6 and 10 weeks after catheter ablation ^a^. Molarity to mass conversion factor for apixaban is 0.460. ^**a**^ The lower and upper borders of the boxes represent the first and third quartiles respectively, the central line represents median, whiskers represent minimum and maximum levels excluding outliers. Circles represent outliers with values between 1.5 and 3 times the interquartile range, the asterisks represent outliers more than 3 times the interquartile range. A single case at baseline had an apixaban concentration of 870 nmol/L. This value is not shown to increase readability of the figure.

Mean apixaban concentrations at baseline and two, six and ten weeks after ablation were 194, 200, 189 and 195 nmol/L respectively. The course of concentrations over time are shown in Fig 1.

Individual apixaban plasma concentrations over time in the 48 patients who had complete sets of four blood samples are depicted graphically in S1 Fig.

The inter-individual (random effect) variance and the intra-individual (residual) variance were estimated as 9004.5 = 94.9^2^ and 3168.5 = 56.3^2^ (nmol/L)^2^, respectively. The overall mean was 194.5 nmol/L, and the corresponding coefficients of variation were thus 94.9/194.5 = 49% and 56.3/194.5 = 29%, respectively.

In the linear regression analysis exploring variables potentially influencing plasma concentrations, we found statistically significant, positive coefficients for increased age, female sex, lower GFR, higher CHA_2_DS_2_-VASc score, use of CYP3A4 and/or P-gp inhibitors and use of amiodarone (Table 2). Adjusting the GFR for age gave substantially the same results (estimate -2.57, 95% CI -3.32 to -1.82, p<0.001). S2 Fig shows the effects of various variables on apixaban concentration.

**Table 2:** Linear mixed effect regression analysis with apixaban plasma concentration (nmol/L) as dependent variable, time point as a categorical covariate and age, sex, renal function, CHA_2_DS_2_-VASc score or concomitant drug treatment one at a time as covariate.

	Regression coefficient		
	Estimate	95% CI	P value
Age (years)	3.56	1.78 to 5.35	<0.001
Female sex	101.8	67.8 to 135.8	<0.001
GFR (mL/min)	-2.52	-3.12 to -1.91	<0.001
CHA_2_DS_2_-VASc score	30.4	20.3 to 40.5	<0.001
Use of CYP3A4 and/or P-gp inhibitor^a^^,^^b^	62.1	28.7 to 95.5	<0.001
Use of amiodarone^c^	60.5	24.8 to 96.3	0.001

Abbreviations: CI = confidence interval; GFR = glomerular filtration rate; CYP3A4 = cytochrome P450 3A4; P-gp = P-glycoprotein

^a^ CYP3A4 and/ or P-gp inhibitors were verapamil, diltiazem, dronedarone and amiodarone

^b^ No use of CYP3A4 and/or P-gp inhibitor as reference

^c^ No use of amiodarone as reference

Only two patients used CYP3A4 and/or P-gp inducers, and this variable was therefore not included in the analysis. Patients using amiodarone had a higher mean CHA_2_DS_2_-VASc score than the group not using amiodarone (2.72 vs. 1.93). We therefore checked whether the effect of CHA_2_DS_2_-VASc scores on apixaban plasma concentrations could be explained by an association between CHA_2_DS_2_-VASc and use of amiodarone by entering the latter as a covariate. However, the coefficient for CHA_2_DS_2_-VASc remained substantially unchanged (it was reduced from 30.4 to 27.5) after this procedure.

**Fig 2:**
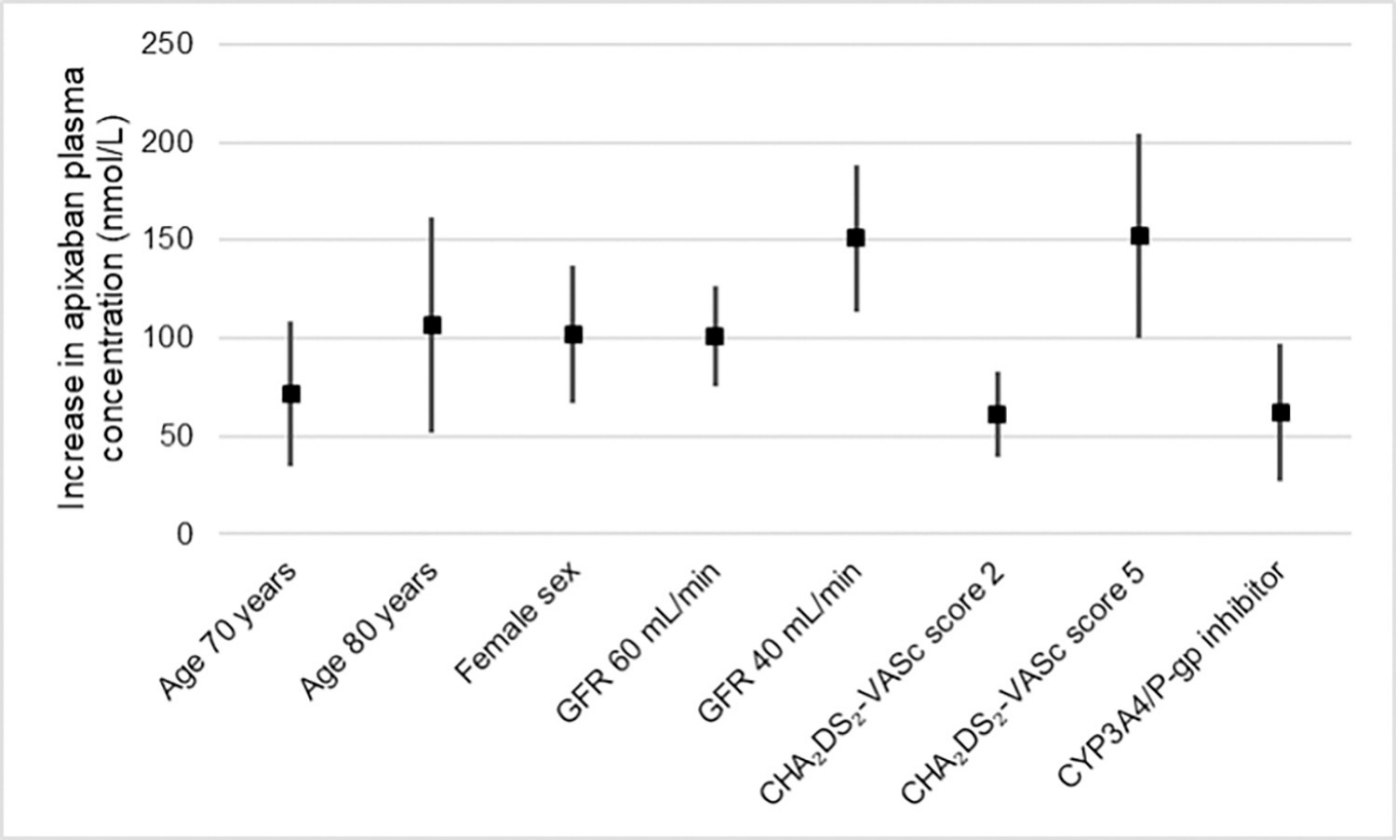
Estimates of effect of various factors on apixaban concentrations. Estimated increases in apixaban plasma concentrations compared to an index patient defined as a 50-year old man with a glomerular filtration rate (GFR) of 100 mL/min and a CHA_2_DS_2_-VASc score of 0, not using any CYP3A4/P-glycoprotein inhibitors. Values are derived from the estimates presented in Table 2. Vertical lines represent the 95% confidence intervals of the estimates.

Overall, sex seemed to play a substantial role on apixaban concentrations. As seen from Table 2, being female resulted in a concentration which was approximately 100 nmol/L higher than men. This effect is approximately equivalent to ageing 30 years or to a reduction in GFR of 40 ml/min. The use of amiodarone or other CYP3A4/P-gp inhibitors had just over half of the effect of the female sex on plasma concentrations of apixaban (Fig 2).

Of the 140 patients who returned the MMAS-8 at baseline, 83 patients (59%) had a score of 8. The lowest score was 3.75. Of the 128 patients who returned the MMAS-8 at the end of the study, 83 patients (65%) had a score of 8. The lowest score was 2.75. There was no correlation between MMAS-8 score and apixaban plasma concentration, neither at baseline (p = 0.45) nor at the end of study (p = 0.15).

## Discussion

The principal findings of the present study was that higher age, female sex, lower GFR, and use of CYP3A4 and/or P-gp inhibitors in general and amiodarone in particular, all were associated with increased plasma concentrations of apixaban. These results correspond well to the findings from similar studies performed in other settings [18–20].

Increasing age leads to several changes in pharmacokinetic processes. Hepatic drug clearance declines with age due to reduced hepatic blood flow and/or reduced metabolic capacity [21]. It seems however that reduced renal function is the most important factor explaining increased levels of apixaban concentrations with increasing age [22]. About 30% of apixaban is eliminated via the renal pathway. Previous studies indicate that apixaban exposure increases modestly with increasing renal impairment, and severe renal impairment may increase the apixaban AUC by approximately 40% [23]. Body weight, lean body mass, liver mass, and organ perfusion are generally lower in women than men. The GFR is also on average about 10% lower in women [24]. These factors may all contribute to the higher levels of apixaban in women.

Increased CHA_2_DS_2_-VASc scores were associated with increased plasma concentrations of apixaban. We expect that this association is explained partly by age being included in the CHA_2_DS_2_-VASc score. A further explanation could be that patients with more comorbidity, particularly those with heart failure, may have a reduced rate of drug metabolism in general and this could in turn lead to higher apixaban concentrations in plasma. The use of amiodarone could perhaps be more frequent in the presence of heart failure or other comorbidities. However, the effect of CHA_2_DS_2_-VASc score on apixaban plasma concentrations was not explained by more use of amiodarone among those with higher CHA_2_DS_2_-VASc scores, as the regression coefficient between CHA_2_DS_2_-VASc score and apixaban concentration remained substantially unchanged when including amiodarone as a covariate.

Patients due for catheter ablation are thoroughly informed about the risk of stroke and the importance of adhering to their anticoagulant medication prior to the procedure. Thus, intra- and inter-individual variability in such a patient group, assumed to be highly adherent to medication and who also scored high on the MMAS-8 adherence scale, would be expected to represent the variability caused by biological factors more closely. Both inter- and intra-individual variability data compared relatively well to the results from previously published studies, which have used indirect measurements for quantifying apixaban in the general apixaban-treated population and a different method for calculating variability [25, 26]. This is somewhat surprising since we expected to find lower inter- and intra-individual variability in a patient group with assumed better adherence than in the population at large, especially when we measured concentrations using precise UHPLC-MSMS technology.

Expected concentrations after intake of standard apixaban doses have been published earlier [9, 27–29]. Two of these sources based on the same patient material state that expected median trough concentration is 103 ng/mL (equivalent to 224 nmol/L) with 5^th^ and 95^th^ percentiles at 41 ng/mL and 230 ng/mL (89 and 500 nmol/L), respectively, in patients with AF treated with 5 mg apixaban twice daily for stroke prevention [27, 28]. A third source cites expected 5^th^ and 95 ^th^ percentiles of trough concentrations in patients treated with apixaban for stroke prevention of 34 ng/mL and 230 ng/mL (74 nmol/L and 500 nmol/L), respectively [9]. Yet another study involving 70 AF patients treated with 5 mg apixaban twice daily reported a median trough concentration of 77 ng/mL (168 nmol/L) with a range from 29–186 ng/mL (63–400 nmol/L) and 10^th^ and 90^th^ percentiles at 47 ng/mL and 121 ng/mL (102 and 263 nmol/L), respectively) [29]. Most patients in our study had trough levels within the ranges of these previous studies, with the majority of apixaban concentrations in the range of 50–500 nmol/L (23–230 ng/mL), S1 Fig.

We found no correlation between MMAS-8 score and apixaban plasma concentration, neither at baseline nor at the end of study. All MMAS-8 questionnaires were answered in private, at home, as opposed to a hospital setting, probably making it easier for patients to answer truthfully [30]. Although there are several instruments for measuring adherence, electronic monitoring being among the most precise, there is still no gold standard [31, 32]. Combining multiple instruments to measure adherence simultaneously could have given a more accurate account of the actual patient adherence. Another reason for lack of correlation between MMAS-8 score and apixaban concentrations could be the very skewed MMAS-8 scores where most patients had optimal compliance and only 11/140 patients and 12/128 patients, respectively, scored less than 6 (i.e., had low adherence) in the first and second MMAS-8 registration. Ultimately, adherence among patients did not very much, and perhaps too little to reliably impact drug concentrations on a group level.

This study has some limitations that should be acknowledged. Patients accepting to participate were highly motivated so the generally high self-reported adherence in this patient group is not surprising. Moreover, participation in the study could on its own merits have led to increased adherence, as patients were sent a reminder to provide a blood sample. These factors may have introduced a bias, with an overestimation of adherence when compared to the general apixaban-treated population. Despite this, the intra- and inter-individual variability in our study corresponded relatively well to those found in the general apixaban-treated population in previous studies [25, 26]. If a therapeutic concentration range for apixaban is defined and future studies confirm our findings of overall intra- and inter-individual variabilities in patients with high adherence, there might be a need for a more liberal approach to measuring patients’ apixaban concentrations than is today’s practice. This might be particularly relevant since we have shown an equally large inter-individual variability in a group assumed to be highly adherent as in the general apixaban-treated population.

Another limitation is that trough levels were calculated using a fixed elimination half-life of 12 hours. This procedure might have introduced a certain degree of imprecision as compared to having obtained the samples exactly at trough. Ideally, we would also have included patients using rivaroxaban, but these were deemed too few in number and were therefore excluded. Two thirds of our patients had incomplete sets of blood tests. We tried to compensate for this by applying a linear mixed model analysis in order to allow all collected plasma concentration data to contribute to the analysis. We also did not analyze for the effect of CYP3A4 and/or P-gp inducers on plasma concentration because there were only two patients on medication classified as such according to the source we used [9], and this number was deemed too small for proper statistical analysis. In addition, the drug in question was prednisolone, which only has a weak enzyme inducing effect in vivo [33]. Finally, it is also a limitation that we did not measure any clinical endpoints in this study. This could be an interesting area of research in future studies, although we anticipate that evaluating the risk of e.g. ischemic stroke or hemorrhage in patients using DOACs undergoing catheter ablation, with a prospective design such as in the present study, would require a very large number of patients.

## Conclusion

Higher age, female sex, lower GFR, unfavorable cerebrovascular risk profile and use of CYP3A4 and/or P-gp inhibitors including amiodarone were all associated with increased plasma concentrations of apixaban as measured by UHPLC-MSMS. We found no correlation between self-reported adherence assessed by the MMAS-8 and apixaban plasma concentration. Intra- and inter-individual variability compared well to previously published data in the general apixaban-treated population. Due to the variations in plasma concentrations demonstrated, and provided that a defined therapeutic range can be established, therapeutic drug monitoring might be warranted to ensure that concentrations are within the target area.

## Supporting information

S1 Fig Slope graph depicting apixaban plasma concentration (nmol/L) over time in 48 patients who had a complete set of blood samples, where each patient is represented by a coloured line. (TIF)

S2 Fig Effects of sex, CYP3A4/P-gp inhibitor treatment, amiodarone treatment, age, GFR and CHA_2_DS_2_-VASc score on apixaban concentrations. (TIF)
