# Supplementary figures and images for: Apixaban plasma concentrations before and after catheter ablation for atrial fibrillation
Source: PLoS One. 2024 Jul 31;19(7):e0308022. doi: 10.1371/journal.pone.0308022 (PMC11290617; doi:10.1371/journal.pone.0308022)

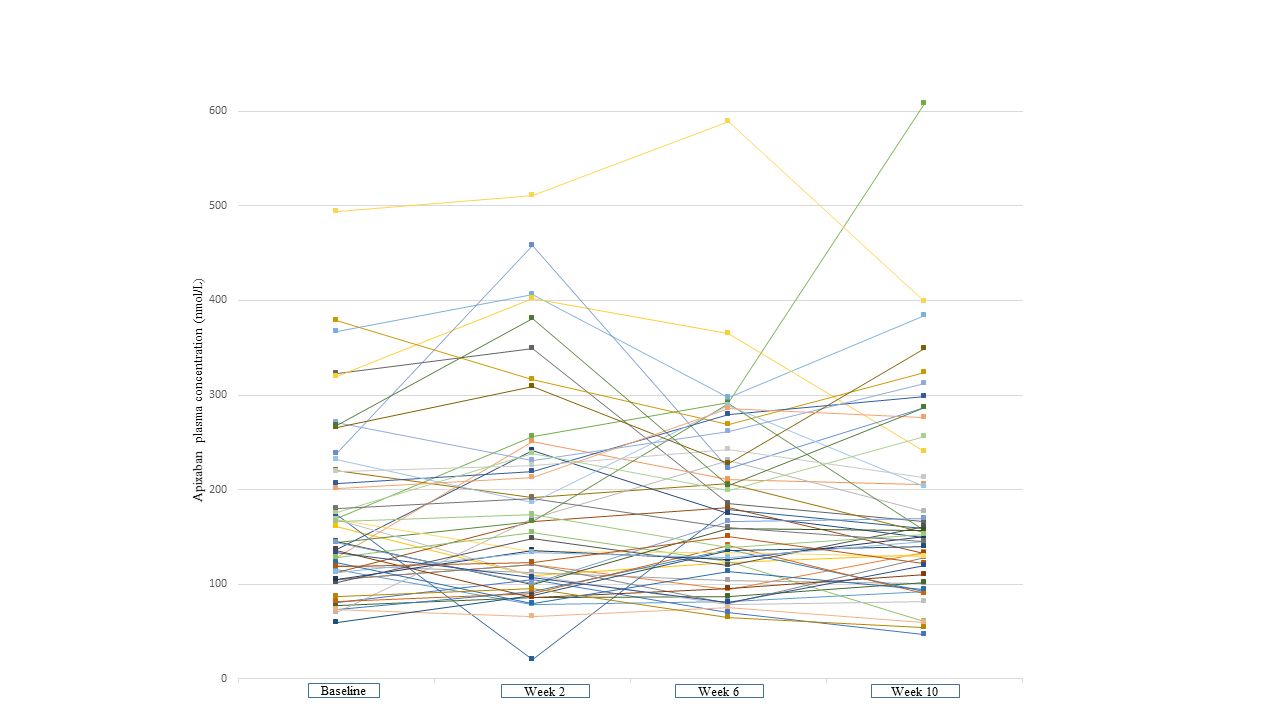

Supplement: S1 Fig — (TIF) [file pone.0308022.s001.tif]

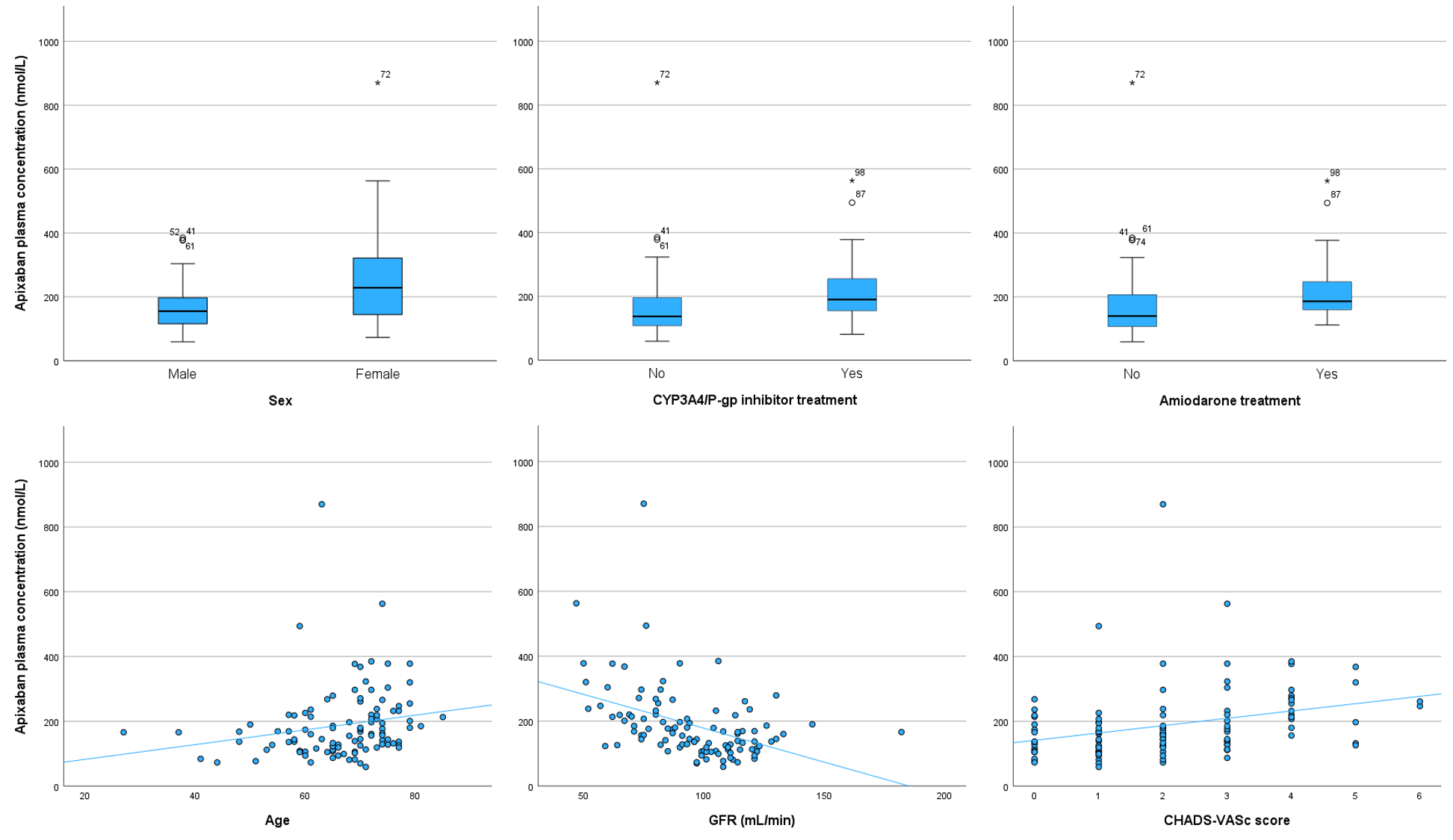

Supplement: S2 Fig — (TIF) [file pone.0308022.s002.tif]
